# Compartment-specific analysis reveals disrupted astrocytic calcium transients in Alzheimer’s mice

**DOI:** 10.21203/rs.3.rs-6682029/v1

**Published:** 2025-06-15

**Authors:** Md Joynal Abedin, Yee Fun Lee, Melinda Zhang, Alyssa N Russ, Dmitry Gerashchenko, Brian J. Bacskai, Ksenia V. Kastanenka

**Affiliations:** Massachusetts General Hospital, Harvard Medical School; Massachusetts General Hospital, Harvard Medical School; Brown University; Massachusetts General Hospital, Harvard Medical School; Department of Psychiatry, Harvard Medical School and Veterans Affairs Boston Healthcare; Massachusetts General Hospital, Harvard Medical School; Massachusetts General Hospital, Harvard Medical School

**Keywords:** Alzheimer’s disease, amyloidosis, multiphoton imaging, calcium imaging, astrocytic compartments

## Abstract

Alzheimer’s disease (AD) is characterized by presence of extracellular amyloid plaques, intracellular tau tangles, and extensive neuronal cell death. In addition to neurons, astrocytes modulate neuronal network activity through tripartite synapses and are increasingly recognized for their involvement in AD pathology. Astrocytic calcium signaling has been implicated in AD pathological processes, including disrupted synaptic transmission, dysregulated glutamate homeostasis, and impaired vascular function via astrocytic endfeet. However, a systematic analysis of calcium dynamics within specific astrocytic compartments has been lacking. Using in vivo multiphoton imaging of Yellow Cameleon 3.6, a genetically encoded calcium indicator targeted to astrocytes in APP/PS1 mice, we analyzed spontaneous calcium transients in cortical astrocytes at 4–6 months of age. We quantified event rate, activity duration, area under the curve (AUC), and peak amplitude across four compartments: soma, processes, microdomains, and endfeet. In APP/PS1 mice, somas exhibited increased activity duration and peak amplitude, while processes and microdomains showed reduced duration, AUC, and amplitude despite higher event rates. Endfeet showed reductions in all parameters. Correlation analysis revealed enhanced astrocyte synchrony in APP/PS1 mice, with distance-dependent correlation decay observed only in nontransgenic controls. Our findings highlight compartment-specific disruptions of astrocytic calcium activity caused by amyloidosis.

## Introduction

Alzheimer’s disease (AD) is the most common form of dementia^1,2^. It is a neurological disorder^3^ with pathological hallmarks of amyloid beta accumulations in the extracellular space and presence of intracellular neurofibrillary tau tangles^4,5^. These pathological hallmarks can lead to neuronal dysfunction^6–8^, synaptic loss^9,10^, neuroinfammation^11–13^, blood-brain barrier leakage^14,15^, and eventual cell death^13,16,17^.

Converging evidence suggests that astrocytes might also contribute to neuronal network impairments associated with AD^18–20^. Astrocytes actively modulate the neuronal network activity in healthy brains^21,22^. Astrocytes form three-way synapses, tripartite synapses, with pre- and post-synaptic neurons^23,24^. Astrocytes express ion channels, including K^+^, Na^+^, and Ca^2+^ distinct from neurons. However, astrocytes do not fire action potentials^25^. Intracellular calcium signaling is fundamental to the physiological processes of astrocytes^25^. Astrocytes are activated by neuronal neurotransmitters and neuromodulators via metabotropic G-protein-coupled receptors and exhibit calcium transients^26^. Activated astrocytes release gliotransmitters that signal to neurons. Therefore, astrocytic calcium signaling plays a fundamental role in astrocyte-neuron communication. These astrocyte-dependent functions are disrupted in patients with AD^27^ and in AD mouse models^28,29^.

Amyloidosis leads to astrocytic hyperactivity in anesthetized mice by activating metabotropic purinergic receptors in vivo^20,30^. Additional reports suggest that astrocytes are hyperactive in the absence of sensory input, yet are hypoactive during sensory-evoked responses^31,32^. These studies offer valuable insights into spontaneous and sensory-evoked calcium signaling in astrocytic somas. However, these studies do not provide detailed information on spontaneous calcium activity of various astrocytic compartments. Astrocytic somas are distinct from primary processes that are adjacent to somas, and microdomains that are adjacent to processes^33^. Astrocytic endfeet ensheathe the vasculature. Astrocytic primary processes and microdomains account for over 80% of the surface area involved in synapse formation, although all compartments, including the soma, could be synaptically connected to neurons^33^. Thus, structural tripartite synapse formation is mostly governed by primary processes and microdomains. While the somas primarily regulates gene expression, astrocytic endfeet are crucial for neurovascular coupling^34^. Therefore, different regions of the astrocytes play distinct roles in maintaining cellular function in a healthy brain. Calcium signaling, essential for cellular function, varies across astrocytic compartments^35^, and aberrations in calcium signaling within distinct astrocytic compartments could have differential effects on Alzheimer’s progression. However, Alzheimer’s field lacks compartment-specific calcium analyses, particularly in mouse models of amyloidosis. Thus, we investigated calcium disruptions in distinct astrocytic compartments in anesthetized APP/PS1 mice.

This study utilized multiphoton imaging of Yellow Cameleon 3.6 (YC3.6), a genetically encoded ratiometric calcium sensor^36^, in anesthetized APP/PS1 mice, an amyloidosis model of AD. YC3.6 was targeted to astrocytes under the GFAP promoter and allowed monitoring spontaneous calcium transients in distinct astrocytic compartments: somas, processes, microdomains and endfeet. We utilized a moderately progressive amyloidosis model. APP/PS1 mice express two mutations: one in human amyloid precursor protein (APP) and second in presenilin 1 (PS1) genes^37^. Presence of both mutations accelerated disease progression evidenced by accumulations of soluble oligomeric Aβ as well as depositions of amyloid plaques around 5–6 months of age, disruptions of astrocytic calcium transients^19,20,32^ and memory impairments.

Calcium transients are often classified as hyperactive or hypoactive, relying on limited binary metrics that may overlook the nuanced dynamics of these signals. To get a comprehensive assessment of astrocytic calcium dynamics, we used five distinct metrics to analyze calcium transients: percentage of active compartments, event rate, activity duration, area under the curve (AUC), and peak amplitude. This approach allowed us to capture the shape and complexity of the calcium transients, rather than constraining their classification to binary states: hyperactive vs hypoactive. We performed the analyses using the five metrics in four distinct astrocytic compartments: somas, processes, microdomains and endfeet. We divided each astrocyte into four compartments aimed to elucidate the differences in calcium dynamics between distinct compartments. Furthermore, we determined aberrations in compartment-specific calcium dynamics in APP/PS1 compared to those in nontransgenic (NTG) mice. This provided a comprehensive characterization of calcium dynamics across entire astrocytes with high resolution in healthy mice and a mouse model of amyloidosis.

We discovered that astrocytic somas exhibited elevated activity duration and higher peak amplitude in APP/PS1 mice compared to those in NTG controls. However, the processes and microdomains showed higher event rate, lower AUC, reduced activity duration and smaller peaks. In contrast, astrocytic endfeet exhibited reduced activity across all measured metrics. We also determined the degree of astrocytic synchrony to elucidate how communication between astrocytes was affected in APP/PS1 mice. We reported that the pairwise correlation (an indicator of synchronization) between astrocytes was increased in APP/PS1 mice. Moreover, an inverse relationship between astrocyte-to-astrocyte distance and pairwise correlation was impaired in APP/PS1 mice compared to that in NTG controls. These findings provide insights into compartment-specific aberrations in calcium activity within astrocytes as a result of amyloidosis.

## Materials and methods

### Experimental animals

This study utilized a transgenic mouse line expressing the Swedish mutation of the amyloid precursor protein and the deltaE9 mutation in presenilin 1 (APPswe/PS1dE9; APP/PS1 mice, stock #034829, The Jackson Laboratory) as well as age-matched nontransgenic littermate controls (NTG mice)^38^. Animals were 4–6 months old and included both males and females. All procedures involving mice were performed with the approval of the Massachusetts General Hospital (MGH) Institutional Animal Care and Use Committees (IACUC, protocol # 2012N000085) and followed the relevant guidelines and regulations, including the ARRIVE guidelines. The mice were housed in a pathogen-free environment with four animals per cage and had access to food and water ad libitum. The facility lighting was maintained on an automated 12-hour light/12-hour dark cycle.

### Stereotaxic injection of adeno-associated viral vectors (AAVs) targeting Yellow Cameleon 3.6

4–6-month-old APP/PS1 and NTG mice were used for astrocytic calcium activity measurements. The animals were anesthetized with isoflurane inhalation (5% induction, 1.75% maintenance), and each were placed into a stereotaxic instrument. The body temperature was maintained with a heating pad during injections. To prevent dryness, eye ointment was carefully applied to the eyes of the mice, and their fur was removed from the scalp using a trimmer. Liquid iodine was then applied to the exposed skin and wiped clean with 70% isopropyl alcohol swabs three times. An incision was made with sterile surgical scissors to expose the skull, and a small burr hole was drilled into the injection site with the following coordinates: anterior-posterior (AP) −1.5 mm, lateral-medial (ML) −1.5 mm, and dorsal-ventral (DV) −0.8 mm. The mice received intracortical viral injections of 3 μL of AAV2/5-gfa2-YC3.6, which specifically targeted Yellow Cameleon 3.6 (YC 3.6, titer = 4×10^12^ vg/ml) to astrocytes in the right somatosensory cortex. The injections were performed using a 33-gauge metal needle and a 10 μl glass syringe controlled by a stereotaxic injector at a rate of 100 nl/min. Following each intracortical injection, the scalp was sutured, and the mice were allowed to recover from anesthesia on a heating pad until they were awake and freely moving. The viral vector was allowed to express for at least four weeks prior to imaging.

### Cranial window installation and multiphoton imaging

After receiving an AAV2/5-gfa2-YC3.6 injection, APP/PS1 and NTG mice underwent cranial window implantation procedures over the injection sites in the right somatosensory cortices. These procedures allowed the monitoring of astrocytic calcium transients using multiphoton microscopy. The animals were anesthetized with isoflurane inhalation (5% induction, 1.75% maintenance), and a round craniotomy was performed using a dental drill. A 5 mm glass coverslip was then placed over the cortex, and the coverslip was secured to the skull with a mixture of Krazy glue and dental cement. A mode-locked titanium/sapphire laser (Mai Tai; Spectra-Physics, Fremont, CA) was used to generate two-photon fluorescence with 860 nm excitation, and three photomultiplier tubes (PMTs) (Hamamatsu Photonics, Japan) were used to detect fluorescence emissions in the range of 380–480 nm, 500–540 nm, and 560–650 nm. A 25X water immersion objective (NA = 1.05, Olympus) was used to image YC3.6-expressing astrocytes. Time-lapse images were acquired at a resolution of 256 × 256 pixels and sampling frequency of 2 μs/pixel. To avoid phototoxicity, laser power was maintained below 50 mW. 150 μl of texas red dextran (70 kDa; 12.5 mg/ml in PBS; Invitrogen) was administered by retroorbital injections to label the vasculature and imaged using multiphoton microscopy through cranial windows. After acquiring multiphoton images, mice were sacrificed using CO_2_ inhalation.

### Image analysis

ImageJ/FIJI (NIH) was used to process timelapse images of compartment-specific YC 3.6-expression within cortical astrocytes. First, each timelapse (250 sec long) was loaded into ImageJ and split into the YFP and CFP channels. Regions of interest (ROIs) containing either somas, processes, microdomains, or endfeet were selected manually. The raw mean grey values for each ROI of the YFP and CFP channels were measured. The YC3.6 ratios were calculated by dividing the emitted fluorescence intensity of YFP by CFP. A custom MATLAB script was used to generate calcium traces (plotted as ΔR/R_o_ over time) for each ROI. To determine the ΔR/R_o_ values, baseline ratio (R_o_) was calculated using the asymmetric least-squares mean smoothing method,^39,40^ which allowed us to find a variable baseline R_o_ without requiring prior information about the peak areas of the signal. The ratio change over the baseline was calculated using the following equation: ΔR/R_o_ = (R − R_o_)/R_o_, where R is instantaneous ratio. After visually inspecting every trace, a threshold of 0.2 was set to exclude optical noise, and the active cell compartment was defined as having activity above this threshold. The cumulative area under the curve and the cumulative activity duration above the threshold was calculated. The cumulative area under the curve and cumulative activity duration was normalized over the 250 second and scaled to a 60 second period. The peak amplitude values were calculated using MATLAB’s findpeaks function, which returned the local maxima of the trace. Local maxima counts were normalized over the 250 second and scaled to a 60 second period which is defined as the event rate in the different astrocytic compartments. Pairwise correlations were calculated for each combination of somas within a timelapse using Pearson’s Linear Correlation Coefficient and were compared between the two groups. To determine the distances between the somas, the centroids of the somas were exported from ImageJ to MATLAB script to calculate the Euclidean distance. Pairwise correlation values between somas were binned over a distance of 10 μm, and a relationship between pairwise correlation and distance was determined.

### Statistical analyses

All statistical analyses were performed using MATLAB (version R2023a). The Shapiro-Wilk normality test was performed to assess the data distribution. Data sets comparing NTG and APP/PS1 were analyzed using a two-sample Student’s t-test. Pearson’s Linear Correlation Coefficient was used to measure the correlation between distance and pairwise correlations. The Kolmogorov-Smirnov (K-S) test was used to compare pairwise correlations between the groups. Statistical significance was set at p < 0.05. * p < 0.05, ** p < 0.01, *** p < 0.001.

## Results

### Calcium transients in the astrocytic soma of APP/PS1 mice exhibited a longer duration and increased amplitude compared to those of NTG controls.

**Figure 1:**
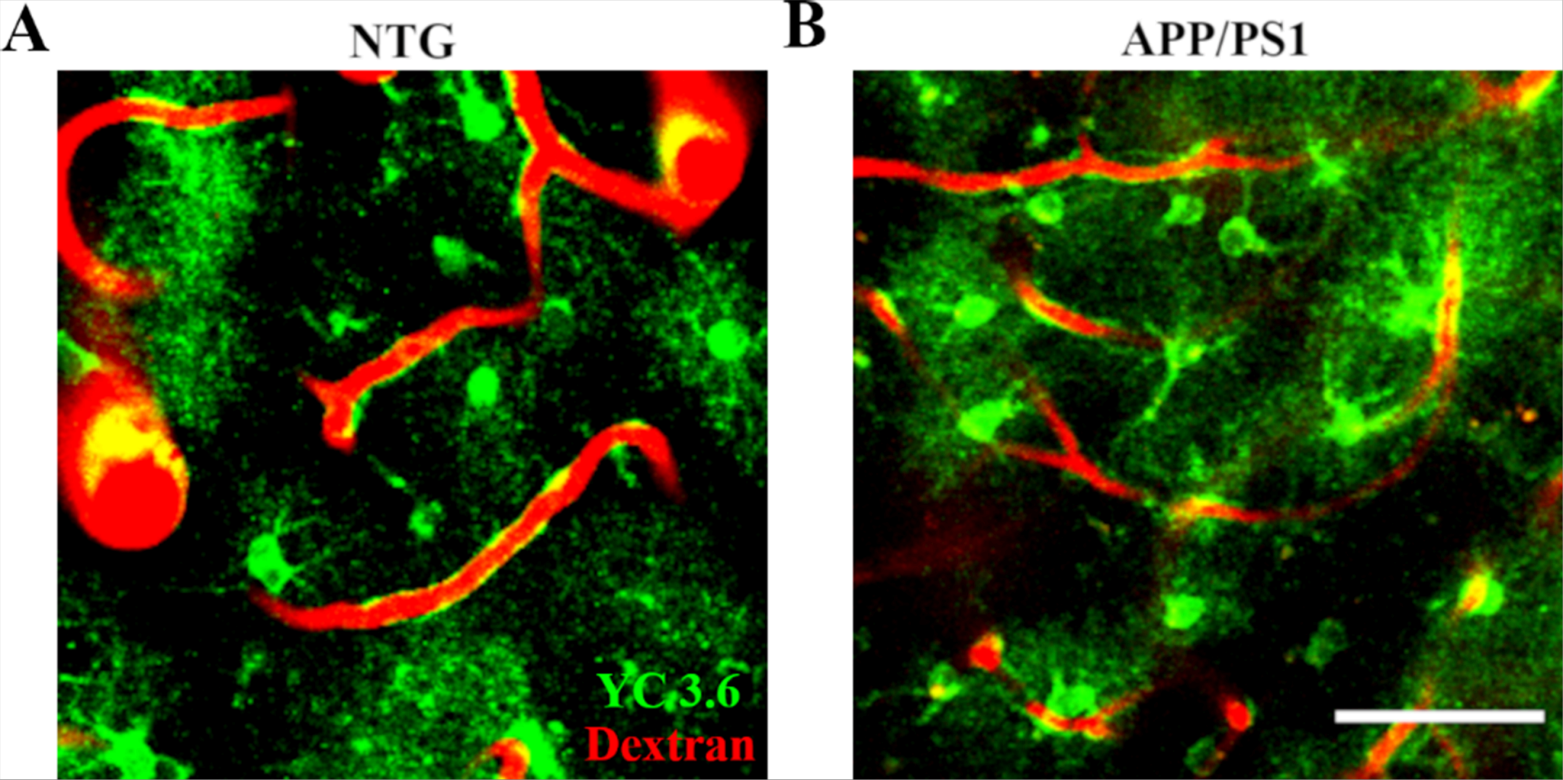
Yellow Cameleon 3.6 (YC3.6) was expressed within different astrocytic compartments in nontransgenic (NTG) and APP/PS1 mice. **(A)**YC3.6 expression (green) in astrocytic soma, processes, microdomains and endfeet in NTG mice. **(B)** YC3.6 expression (green) in astrocytic soma, processes, microdomains and endfeet in APP/PS1 mice. Dextran red shows the blood vessels. Scale bar, 50 μm.

We investigated whether cortical astrocytes exhibited altered spontaneous calcium activity in APP/PS1 mice compared to astrocytes in NTG littermate controls. Astrocytes were targeted with the ratiometric calcium indicator Yellow Cameleon 3.6 (YC 3.6) via the GFAP promoter in the somatosensory cortex. We assessed the expression of YC 3.6 and confirmed its presence in the somas, processes, microdomains, and endfeet of both NTG and APP/PS1 mice (Fig. 1A, B). Astrocytic calcium transients were visualized using multiphoton microscopy in anesthetized mice. To develop a comprehensive understanding of calcium activity at subcellular resolution, we selected the regions of interest (ROIs) pertaining to distinct astrocytic compartments, including somas, processes, microdomains, as well as endfeet. Astrocytic calcium transients were analyzed separately in each compartment.

**Figure 2:**
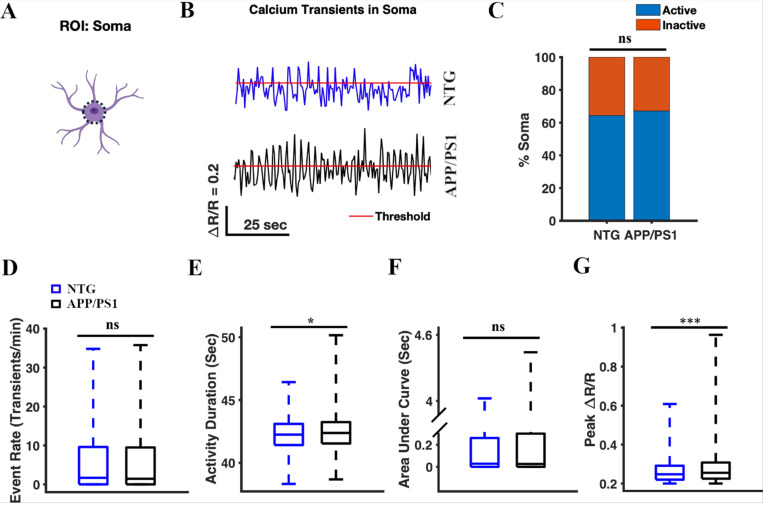
Astrocytic calcium activity in the soma of APP/PS1 mice showed elevated activity duration and higher amplitude of calcium events compared to NTG controls. **(A)** Astrocyte schematic outlining the region of interest (ROI: soma) where activity was measured. **(B)** Representative traces of astrocytic Ca^2+^ activity in the soma of the NTG (blue) and APP/PS1 (black) mice. Red line represents threshold. **(C)** Percentage of active somas in NTG control and APP/PS1 mice. The chi-square test was used to measure statistical significance. Chi-square (χ^2^) = 0.83, p = 0.37. NTG, n = 480 somas, APP/PS1, n = 448 somas. **(D)** Box plot of the event rate of astrocytic Ca^2+^ activity in the soma. The event rates were calculated using a ΔR/R threshold of 0.2 (see materials and methods). A two-sample Student’s t-test was used to measure the statistical significance, p = 0.93. **(E)** Box plot of the area under the curve of astrocytic Ca^2+^ activity in somas. A two-sample Student’s t-test was used to measure the statistical significance, p = 0.013. **(F)** Box plot of the cumulative duration of astrocytic Ca^2+^ activity in the somas. A two-sample Student’s t-test was used to measure the statistical significance, p = 0.01. **(G)** Box plot of ΔR/R peak values from astrocytic Ca^2+^ activity in somas. A two-sample Student’s t-test was used to measure statistical significance, p = 1.37×10^−7^.

To monitor calcium transients in astrocytic somas we manually selected the somas as ROIs (Fig. 2A). A total of 480 somas were identified across 64 timelapses in seven APP/PS1 mice. 448 somas were identified across 64 timelapses in seven NTG mice. We generated astrocytic calcium activity traces, calcium transients, measured as ΔR/R_o_ over time for each ROI, where ΔR is the difference between the R ratio of YC 3.6 and R_o_, while R_o_ is the baseline ratio (Fig. 2B). ΔR/R_o_ traces were thresholded and calcium transient analysis was performed (see Methods for more details). We determined percentages of active and inactive somas. We found that the percentages of active somas were comparable in NTG and APP/PS1 mice (64.38% active somas in NTG mice and 67.19% active somas in APP/PS1 mice, Chi-square test, p = 0.37) (Fig. 2C). We also analyzed calcium events by calculating event rate, cumulative activity duration, cumulative area under the curve, and peak amplitude. The cumulative activity duration of astrocytic somas was significantly higher in APP/PS1 mice than the cumulative activity duration in NTG mice (42.37 [41.54, 43.24] seconds and 42.24 [41.41, 43.1] seconds, respectively, median [Q1 (1st Quartile), Q3 (3rd Quartile)], two-sample student’s t-test p = 0.013) (Fig. 2E). Similarly, the peak amplitude values of astrocytic somas in APP/PS1 mice were significantly higher than those in NTG mice (0.26 [0.22, 0.31] and 0.25 [0.22, 0.29] respectively, two-sample Student’s t-test p = 1.37×10^−7^) (Fig. 2G). In contrast, there was no significant difference in the event rate of astrocytic somas (1.44 [0, 9.48] transients/min and 1.68 [0, 9.6] transients/min, respectively, two-sample Student’s t-test, p = 0.93) between APP/PS1 and NTG mice (Fig. 2D). Furthermore, there was no significant difference in the cumulative area under the curve of astrocytic somas (0.03 [0, 0.3] s and 0.03 [0, 0.26] s, respectively, two-sample Student’s t-test, p = 0.06) between APP/PS1 and NTG mice (Fig. 2F). Based on these results, we concluded that astrocytic soma in APP/PS1 mice displayed calcium signals with extended duration and heightened magnitude when compared to those in NTG counterparts (Fig. 2B).

### Calcium transients in the astrocytic processes of APP/PS1 mice exhibited a higher frequency, shorter duration, decreased intensity (AUC), and reduced amplitude compared to those of NTG controls.

**Figure 3:**
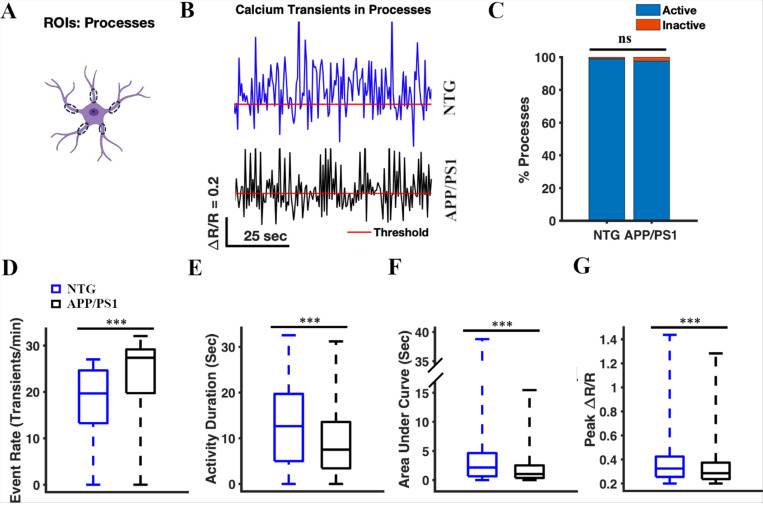
Calcium transients in astrocytic processes of APP/PS1 mice display a higher frequency, shorter duration, lower intensity (AUC), and reduced amplitude compared to those in NTG controls. **(A)** Astrocyte schematic outlining the regions of interest (processes) where the activity was measured. **(B)** Representative traces of astrocytic Ca^2+^ activity in the processes from NTG (blue) and APP/PS1 (black) mice. Red line represents threshold. **(C)** The percentage of active processes in NTG controls and APP/PS1 mice. The chi-square test was used to measure statistical significance. Chi-square (χ^2^) = 2.13, p = 0.14. NTG, n = 423 processes; APP/PS1, n = 257 processes. **(D)** Box plot of the event rate based on astrocytic Ca^2+^ activity. The event rates were calculated using a ΔR/R threshold of 0.2. A two-sample Student’s t-test was used to measure statistical significance, p = 8.09×10^−5^. **(E)** Box plot of the area under the curve from astrocytic Ca^2+^ activity in the processes. A two-sample Student’s t-test was used to measure statistical significance, p = 1.87×10^−8^. **(F)** Box plot of the cumulative activity duration from astrocytic Ca^2+^ activity in the processes. A two-sample Student’s t-test was used to measure statistical significance, p = 3.27×10^−9^. **(G)** Box plot of ΔR/R peak values from astrocyte Ca^2+^ activity in the processes. A two-sample Student’s t-test was used to measure statistical significance, p= 1.37×10^−7^.

We analyzed spontaneous calcium transients in the astrocytic processes (Fig. 3). We confirmed the YC 3.6 expression in the astrocytic processes (Fig. 1), and manually selected the processes as regions of interest (ROIs) (Fig. 3A). We identified 423 processes across 64 timelapses in seven NTG mice, and 257 processes across 64 timeplases in seven APP/PS1 mice. We analyzed the calcium traces (ΔR/R_o_) for each process ROI (Fig. 3B). The percentages of active processes were high and comparable in NTG and APP/PS1 mice (99.05% active in NTG mice and 97.67% processes active in APP/PS1 mice, Chi-square test, p = 0.14) (Fig. 3C). The cumulative activity duration in astrocytic processes was significantly lower in APP/PS1 mice than that in NTG mice (7.5 [3.41, 13.57] seconds and 12.65 [5.0, 19.71] seconds, respectively, two-sample student’s t-test p = 3.27×10^−9^) (Fig. 3E). Similarly, the cumulative area under the curve was significantly lower in APP/PS1 mice than in NTG controls (1.04 [0.37, 2.52] s and 0.25 [0.32, 0.42] s, respectively, two-sample Student’s t-test p = 1.87×10^−8^) (Fig. 3F). The peak amplitude values of astrocyte processes in APP/PS1 mice were significantly lower than those in NTG mice (0.29 [0.24, 0.37] and 0.25 [0.32, 0.42], respectively, two-sample student’s t-test p = 8.09×10^−5^) (Fig. 3G). However, the event rate of astrocytic processes was significantly higher in APP/PS1 mice than that in NTG mice (27.36 [19.74, 29.16] transients/min and 19.68 [13.26, 24.63] transients/min, respectively, two-sample Student’s t-test p = 8.09×10^−5^) (Fig. 3D). These findings suggested that calcium transients in astrocytic processes of APP/PS1 mice exhibited increased frequency, shorter duration, lower intensity (AUC), and smaller amplitude compared to astrocytic calcium transients in NTG controls (Fig. 3B).

### Calcium transients in the astrocytic microdomains of APP/PS1 mice exhibited higher frequency, shorter duration, decreased intensity, and reduced amplitude compared to those of NTG controls.

**Figure 4:**
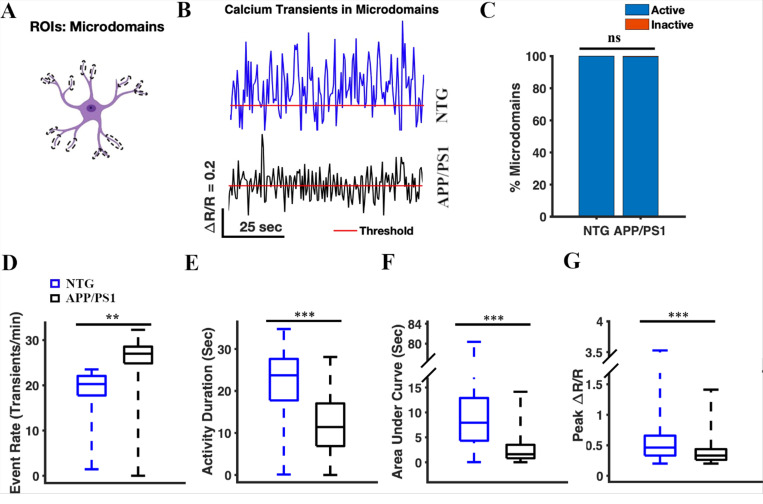
Calcium transients in astrocytic microdomains of APP/PS1 mice, occur more frequently, with shorter duration, lower intensity (AUC), and diminished amplitude compared to those in NTG controls. **(A)** Astrocyte schematic outlining the region of interest (microdomains) where activity was measured. **(B)** Representative traces of astrocytic Ca^2+^ activity in the processes from NTG (blue) and APP/PS1 (black) mice. Red line represents threshold. **(C)** The percentage of active microdomains in NTG and APP/PS1 mice. The chi-square test was used to measure statistical significance. Chi-square (χ^2^) = 2.90, p = 0.09. NTG, n = 799 microdomains; APP/PS1, n = 276 microdomains. **(D)** Box plot of the event rate from astrocytic Ca^2+^ activity in microdomains. The event rates were calculated using a ΔR/R threshold of 0.2. A two-sample Student’s t-test was used to measure the statistical significance, p = 0.004. **(E)** Box plot of the area under the curve of astrocytic Ca^2+^ activity in microdomains. A two-sample Student’s t-test was used to measure statistical significance, p = 2.49×10^−43^. **(F)** Box plot of the cumulative activity duration from astrocytic Ca^2+^ activity in the microdomains. A two-sample Student’s t-test was used to measure statistical significance, p = 2.97×10^−66^. **(G)** Box plot of ΔR/R peak values from astrocytic Ca ^2+^ activity in microdomains. A two-sample Student’s t-test was used to measure statistical significance, p= 8.83×10^−246^.

We analyzed spontaneous calcium transients in the astrocytic microdomains (Fig. 4). First, we confirmed that YC 3.6 expression was present in the astrocytic microdomains (Fig. 1), and manually selected the microdomains as regions of interest (ROIs) (Fig. 4A). We identified 799 microdomains across 64 timelapses in seven NTG mice, and 276 microdomains across 64 timelapses in seven APP/PS1 mice. We analyzed the calcium traces (ΔR/R) for each microdomain ROI (Fig. 4B). We found that all or nearly all microdomains were active in NTG and APP/PS1 mice (100% active in NTG mice and 99.64% active in APP/PS1 mice) (Fig. 4C). The percentage of active microdomains did not differ significantly between NTG and APP/PS1 mice (Chi-square test, p = 0.09) (Fig. 4C). The cumulative activity duration in astrocytic microdomains was significantly lower in APP/PS1 mice than in NTG mice (11.41 [6.87, 17.03] s and 23.72 [17.75, 27.62] s, respectively, two-sample Student’s t-test p = 2.97×10^−66^) (Fig. 4E). Similarly, the cumulative area under the curve was significantly lower in APP/PS1 mice than that in NTG controls (1.62 [0.8, 3.51] s and 7.94 [4.32, 12.9] s, respectively; two-sample Student’s t-test, p = 2.49×10^−43^) (Fig. 4F). The peak amplitude values of astrocyte microdomains in APP/PS1 mice were significantly lower than those in NTG mice (0.33 [0.26, 0.44] and 0.46 [0.33, 0.66], respectively, two-sample student’s t-test p = 8.83×10^−246^) (Fig. 4G). In contrast, the event rate of astrocytic microdomains was significantly higher in APP/PS1 mice than that in NTG mice (27 [24.86, 28.56] transients/min and 20.28 [17.76, 22.08] transients/min, respectively; two-sample Student’s t-test p = 0.004) (Fig. 4D). Taken together, these results indicated that calcium transients in astrocytic microdomains occurred more frequently, had shorter durations, lower intensity (AUC), and smaller amplitude in APP/PS1 mice compared to astrocytic calcium transients in NTG controls (Fig. 4B).

### Calcium transients in the astrocytic endfeet of APP/PS1 mice exhibited lower frequency, shorter duration, decreased intensity (AUC), and reduced amplitude compared to those of NTG controls.

**Figure 5:**
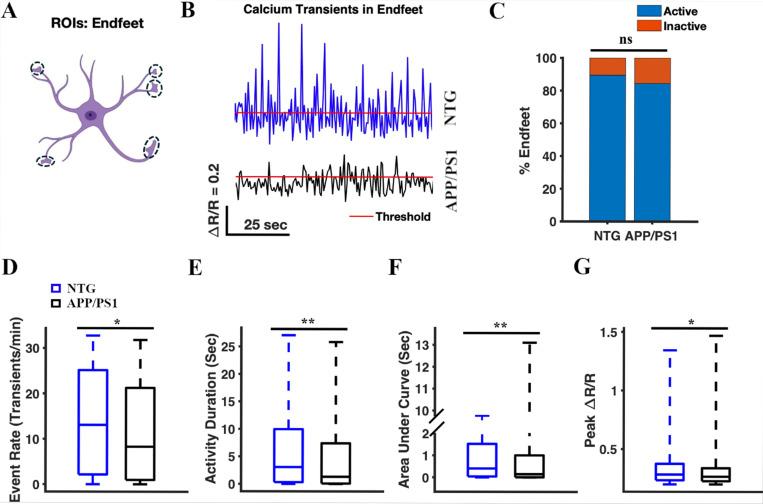
Calcium transients in the astrocytic endfeet of APP/PS1 mice, show a lower frequency, reduced duration, decreased intensity (AUC), and smaller amplitude compared to NTG controls. **(A)** Astrocyte schematic outlining the region of interest (endfeet) where activity was measured. **(B)** Representative traces of astrocytic Ca^2+^ activity in the endfeet of NTG (blue) and APP/PS1 (black) mice. Red line represents threshold. **(C)** The percentage of active microdomains in NTG and APP/PS1 mice. The chi-square test was used to measure statistical significance. Chi-square (χ^2^) = 2.41, p = 0.12. NTG, n = 210 endfeet; APP/PS1, n = 212 endfeet. **(D)** Box plot of the event rate from astrocytic Ca^2+^ activity in the endfeet. The event rates were calculated using a ΔR/R threshold of 0.2. A two-sample Student’s t-test was used to measure the statistical significance, p = 0.01. **(E)** Box plot of the area under the curve of astrocytic Ca^2+^ activity in the endfeet. A two-sample Student’s t-test was used to measure the statistical significance, p = 0.005. **(F)** Box plot of the cumulative activity duration from astrocytic Ca^2+^ activity in the endfeet. A two-sample Student’s t-test was used to determine the statistical significance, p = 0.003. **(G)** Box plot of ΔR/R peak values from astrocytic Ca^2+^ activity in the endfeet. A two-sample Student’s t-test was used to measure the statistical significance, p = 0.02.

Next, we analyzed spontaneous calcium transients in astrocytic endfeet (Fig. 5) in an analogous manner. First, we confirmed YC 3.6 expression in astrocytic endfeet (Fig. 1), and manually selected the endfeet as regions of interest (ROIs) (Fig. 5A). We identified 210 endfeet across 64 timelapses in seven NTG mice. 212 endfeet across 64 timelapses were identified in seven APP/PS1 mice. We analyzed the calcium traces (ΔR/R) for each endfoot ROI (Fig. 5B). The percentages of active endfeet were comparable in NTG and APP/PS1 mice (89.52% active endfeet in NTG mice and 84.43% active endfeet in APP/PS1 mice, chi-square test, p = 0.12) (Fig. 5C). The event rate of calcium transients in astrocytic endfeet of APP/PS1 mice was significantly lower than that in NTG mice (1.31 [0.08, 7.41] transients/min and 3.06 [0.31, 9.96] transients/min, respectively, two-sample Student’s t-test p = 0.003) (Fig. 5D). The cumulative activity duration in astrocytic endfeet was also significantly lower in APP/PS1 mice than that in NTG mice (1.31 [0.08, 7.41] s and 3.06 [0.31, 9.96] s, respectively; two-sample Student’s t-test, p = 0.003) (Fig. 5E). Similarly, the cumulative area under the curve was significantly lower in APP/PS1 mice than in NTG controls (0.15 [0.01, 1.01] s and 0.4 [0.04, 1.53] s, respectively; two-sample Student’s t-test, p = 0.005) (Fig. 5F). Finally, the peak amplitude values of astrocytic endfeet in APP/PS1 mice were significantly lower than those in NTG mice (0.27 [0.23, 0.34] and 0.28 [0.24, 0.38], respectively; two-sample Student’s t-test p = 0.02) (Fig. 5G). Based on these findings, we concluded that calcium transients in astrocytic endfeet of APP/PS1 mice occurred less frequently, had shorter durations, lower intensity (AUC), and smaller peak amplitude compared to those in NTG controls (Fig. 5B).

### The inverse relationship between pairwise correlation of astrocytic activity and astrocyte-to-astrocyte distance was impaired in APP/PS1 mice.

**Figure 6:**
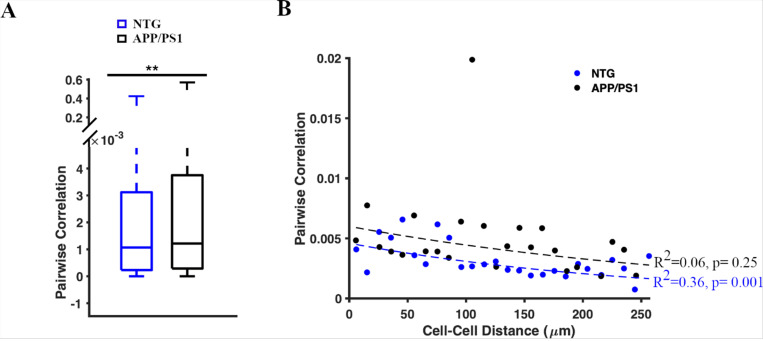
Pairwise correlation of astrocytic Ca^2+^ activity in somas. **(A)** Box plot of pairwise correlations (rho-values) between each pair of somas. NTG, n = 2802 soma pairs; APP/PS1, n = 2613 soma pairs. The Kolmogorov–Smirnov (K-S) test was used to measure statistical significance, p = 0.002. **(B)** Scatter plot representing the relationship between the distance between two somas and their pairwise correlation in NTG (blue) and APP/PS1 (black) mice. Dashed lines show the linear regression of the pairwise correlation between soma pairs. Pearson’s Linear Correlation Coefficient was used to measure statistical significance. R^2^ = 0.06, p=0.25 in APP/PS1 mice and R^2^ = 0.36, p=0.001 in NTG controls.

Finally, we investigated the degree of synchronization in calcium activity of astrocytic somas in NTG and APP/PS1 mice. We calculated pairwise correlations as a measure of somal pair synchronization for each of the 64 timelapses (n = 2808 soma pairs in seven NTG mice; n = 2613 soma pairs in seven APP/PS1 mice). The pairwise correlations between soma pairs were significantly higher in APP/PS1 mice than those in NTG mice (1.2 [0.29, 3.8] × 10^−3^ and 1.07 [0.2, 3.12] × 10^−3^, the Kolmogorov Smirnov (K-S) test, p = 0.002) (Fig. 6A). Thus, astrocytic somas were more synchronized in APP/PS1 mice (Fig. 6A, B). Notably, when determining the effect of distances between two somas on their pairwise correlations, we found that NTG mice followed an inverse relationship between cell-to-cell distances and pairwise correlations. This indicated that as the distance between two somas increased, their pairwise correlation decreased (Pearson’s Linear Correlation Coefficient, R^2^ = 0.36, p = 0.001 in NTG controls.) (Fig. 6B). However, this relationship was absent in APP/PS1 mice (Pearson’s Linear Correlation Coefficient, R^2^ = 0.06, p = 0.25) (Fig. 6B). This loss of statistical significance indicated that the effect of distance on the pairwise correlation of astrocytes was impaired in APP/PS1 mice. This could be due to astrocytes being further apart in the presence of plaques and/or presence of oligomeric Aβ^41^.

## Discussion

Astrocytic calcium activity plays an essential role in brain function and behavior, making it a key area of research in AD field in recent years^42,43^. Astrocytic calcium signaling allows astrocytes to communicate with neurons and effectively regulate synaptic transmission^26,42^. Astrocytic endfeet wrap around blood vessels to regulate vascular coupling^29^. Astrocytic calcium transients can arise spontaneously without neuronal activity or be induced by neurotransmitters released during synaptic activity^44,45^. Genetically encoded calcium indicators can be targeted to astrocytes specifically. High-resolution imaging techniques allow imaging astrocytes to study their calcium activity in vivo. Using the ratiometric calcium indicator YC3.6 and in vivo multiphoton imaging, we were able to elucidate astrocytic calcium dynamics within distinct compartments in APP/PS1 mice. Compartment-specific astrocytic calcium transients were analyzed using five distinct analysis metrics: percentage of active compartments, the event rate, area under the curve (AUC), activity duration, and peak amplitude. We reported that astrocytic calcium signaling showed distinct compartment-specific aberrations in APP/PS1 mice compared to those in NTG controls. Calcium transients in the astrocytic soma of APP/PS1 mice lasted longer and had increased amplitude. Astrocytic microdomains and processes exhibited increased frequency but shortened duration, reduced intensity, and amplitude. Astrocytic endfeet exhibited lower-frequency calcium transients with shorter duration, reduced intensity (AUC), and lower amplitude. Additionally, an inverse relationship between astrocytic activity correlations and cell-to-cell distance seen in NTG controls was absent in APP/PS1 mice, suggesting disrupted spatial activity patterns.

Prior literature reported astrocytic somas exhibiting higher calcium event frequencies in 6–9-month-old APP/PS1 mice expressing GCaMP7 compared to those in NTG littermate controls^32^. Similarly, imaging of the small-molecule calcium chelator BAPTA showed elevated spontaneous calcium activity in 6–8-month-old APP/PS1 mice compared to rare events in NTG controls^20^. However, astrocytic somas in our 4–6-month-old APP/PS1 mice did not exhibit significant increases in event rate as shown in present study, likely due to younger age of mice, since older mice with higher plaque burden might experience greater disruptions in astrocytic calcium transients. Consistent with previous fndings^20^, we observed higher calcium transient amplitudes in astrocytic somas of APP/PS1 mice. We further demonstrated that astrocytic somas exhibited prolonged calcium durations in APP/PS1 mice compared to those in NTG controls. Somal proximity to plaques is not expected to affect the results because previous studies showed that Aβ-plaque proximity did not have a direct effect on either the frequency or the duration of astrocytic calcium transients^46^, The cell-wide astrocytic calcium dysregulation was not directly related to amyloid plaque proximity^47^.

Astrocytic processes and microdomains were reported to exhibit increased calcium event frequencies in 6–9-month-old APP/PS1 mice expressing GCaMP7^20^. Similarly, our 4–6-month-old APP/PS1 mice showed a marked increase in event rates within these astrocytic compartments. Despite increased event frequency, astrocytic processes and microdomains displayed reduced calcium activity duration, intensity (AUC), and amplitude in young APP/PS1 mice compared to those measures in NTG controls.

We also observed that astrocytic endfeet exhibited reduced calcium event frequency, shorter durations, lower intensity (AUC), and diminished amplitude in anesthetized APP/PS1 mice at 4–6 months. Studies monitoring calcium transients within astrocytic endfeet in anesthetized APP/PS1 mice remain limited.

We also reported an inverse relationship between astrocyte distance and pairwise correlation in NTG mice that was impaired in APP/PS1 mice. Our data is consistent with prior literature confirming that astrocytes showed stronger correlation in activity in APP/PS1 mice compared to astrocytes in NTG^20^. However, we acknowledge that YC3.6 virus targets a limited number of astrocytes in proximity to the injection cite. Therefore, YC3.6 expression may influence cell spacing and signaling correlations. Furthermore, general anesthesia has been shown to alter astrocytic calcium transients, including their synchronized activity^48^. Thus, future studies in absence of anesthesia will be necessary.

Recent work reported that astrocytic soma and primary processes are hyperactive, whereas microdomains and astrocytic endfeet are hypoactive in plaque-bearing, non-anesthetized APP/PS1 mice^47^. These studies tended to report dysregulation of astrocytic calcium transients in the context of AD by analyzing astrocytic calcium transients using limited metrics in discrete compartments. Thus, our comprehensive compartment-specific analysis expands upon these findings.

The observed reduction in calcium activity across several metrics within astrocytic processes, microdomains, and endfeet adds complexity to the established narrative of astrocytic calcium hyperactivity in amyloidosis mouse models. This nuanced pattern suggests that, while soma may exhibit elevated calcium signaling, other astrocytic compartments show diminished activity, indicating a more intricate and compartment-specific alteration in astrocytic function in AD mice. Elevated somatic calcium signaling can dysregulate gene expression by activating calcium-dependent transcription factors such as Nuclear factor of activated T cells (NFAT), cyclic adenosine monophosphate response element binding protein (CREB), and Nuclear factor kappa B (NF-κB). Each of these molecular players has a role in neuroinflammation and cellular stress responses as part of Alzheimer’s progression^49–52^. This dysregulation can promote the expression of genes linked to neuroinflammation, astrogliosis, and oxidative stress, exacerbating AD pathology^52,53^. Elevated calcium in soma may also impair astrocytic functions, such as synaptic support and metabolic regulation, further contributing to disease progression^54^. From an epigenetic perspective, aberrant calcium signaling in astrocytic soma can influence histone modifications and DNA methylation patterns, which may lead to the persistent activation of inflammatory pathways and altered gene expression profiles in AD. For example, elevated calcium can activate the NF-κB pathway, which in turn may lead to changes in histone acetylation and methylation that promote the expression of pro-inflammatory genes, contributing to chronic neuroinflammation in AD^55,56^. Additionally, disrupted astrocytic calcium signaling in soma can impact the epigenetic regulation of genes involved in synaptic plasticity and neuroprotection, potentially impairing the brain’s ability to cope with beta-amyloid accumulations^57^. These epigenetic modifications could play a crucial role in the long-term dysregulation of astrocyte function, contributing to the progressive nature of AD^58^.

Reduced calcium signaling in astrocytic processes and microdomains could disrupt neuronal network function via tripartite synapses^33,59^. Astrocytes play a critical role in regulating synaptic activity, neurotransmitter uptake, and gliotransmission through calcium signaling^60^. Impaired astrocytic calcium dynamics could lead to decreased synaptic plasticity, excitotoxicity, and neuronal loss in AD^54,61^. Consistent with this idea, reduced calcium signaling in astrocytic microdomains impaired glutamate uptake, exacerbating excitotoxic damage^54^. Additionally, diminished calcium activity might reduce gliotransmitter release, disrupting synaptic coordination and cognitive function^45^.

Reduced calcium signaling in astrocytic endfeet was shown to disrupt neurovascular coupling, impairing the regulation of blood flow in response to neuronal activity^62,63^. Astrocytic endfeet normally release vasoactive molecules to regulate blood vessel dilation, but in AD, amyloid-beta (Aβ) accumulation and inflammation hindered this function, leading to cerebral hypoperfusion and inadequate nutrient delivery to neurons^64^. This dysfunction contributed to cognitive decline by disrupting the proper vascular response and accumulating metabolic waste products^65,66^. Astrocytic calcium signaling played a critical role in coordinating neurovascular responses and supporting astrocyte-neuron interactions, which were essential for regulating blood flow and maintaining vascular health^60,67^. Beyond its role in neurovascular coupling, astrocytic endfeet dysfunction could also compromise glymphatic clearance, a perivascular waste removal system essential for brain homeostasis^68^. Aquaporin-4 (AQP4), primarily localized to astrocytic endfeet, facilitates interstitial fluid flow along perivascular routes, aiding in the clearance of Aβ and other neurotoxic metabolites^69^. AQP4 is also critical for downstream calcium signaling^70^. In AD, AQP4 mislocalization or dysfunction disrupts glymphatic flow, leading to impaired Aβ clearance, heightened neuroinflammation, and progressive cognitive decline^71,72^. This underscores the critical role of astrocytic calcium signaling not only in regulating vascular function but also in supporting waste clearance pathways vital for maintaining brain health.

In conclusion, our study revealed compartment-specific alterations in astrocytic calcium signaling in APP/PS1 mice, highlighting a complex pattern of aberrant activity across different astrocytic regions. While somatic calcium signaling was elevated; processes, microdomains, and endfeet mostly exhibited reduced activity, suggesting a nuanced compartment specific disruption of astrocytic function in Alzheimer’s disease. These changes in calcium dynamics may contribute to the dysregulation of neuronal networks, synaptic function, and neurovascular coupling, ultimately exacerbating disease pathology. Our findings underscored the importance of a detailed, compartment-specific analysis of astrocytic calcium signaling in AD and provided new insights into how these disruptions may contribute to disease progression, offering potential targets for therapeutic intervention.

## Data Availability

The data that were either generated or analyzed during this study can be found in this published article. All datasets that are not included are available from the corresponding author upon request.

## References

[1] Arvanitakis Z., Shah R. C. & Bennett D. A. Diagnosis Manage. Dementia: Rev. JAMA 322, 1589–1599 (2019). 10.1001/jama.2019.4782 PMC7462122 31638686

[2] Jahn H. Memory loss in Alzheimer’s disease. Dialogues Clin. Neurosci. 15, 445–454 (2013). 24459411 10.31887/DCNS.2013.15.4/hjahn PMC3898682

[3] Lane C. A., Hardy J. & Schott J. M. Alzheimer’s disease. Eur. J. Neurol. 25, 59–70 (2018). 28872215 10.1111/ene.13439

[4] Selkoe D. J. & Hardy J. The amyloid hypothesis of Alzheimer’s disease at 25 years. EMBO Mol. Med. 8, 595–608 (2016). 27025652 10.15252/emmm.201606210 PMC4888851

[5] Hyman B. T. National Institute on Aging-Alzheimer’s Association guidelines for the neuropathologic assessment of Alzheimer’s disease. Alzheimers Dement. 8, 1–13 (2012). 22265587 10.1016/j.jalz.2011.10.007 PMC3266529

[6] Mattson M. P. Involvement of GABAergic interneuron dysfunction and neuronal network hyperexcitability in Alzheimer’s disease: Amelioration by metabolic switching. Int. Rev. Neurobiol. 154, 191–205 (2020). 32739004 10.1016/bs.irn.2020.01.006

[7] Palop J. J. & Mucke L. Amyloid-β–induced neuronal dysfunction in Alzheimer’s disease: from synapses toward neural networks. Nat. Neurosci. 13, 812–818 (2010). 20581818 10.1038/nn.2583 PMC3072750

[8] Santos S. F., Pierrot N. & Octave J. N. Network excitability dysfunction in Alzheimer’s disease: insights from in vitro and in vivo models. Rev. Neurosci. 21, 153–171 (2010). 20879690 10.1515/revneuro.2010.21.3.153

[9] Bhembre N., Bonthron C. & Opazo P. Synaptic Compensatory Plasticity in Alzheimer’s Disease. J. Neurosci. 43, 6833–6840 (2023). 37821232 10.1523/JNEUROSCI.0379-23.2023 PMC10573755

[10] Tönnies E., Trushina E. O. & Stress Synaptic Dysfunction, and Alzheimer’s Disease. J. Alzheimers Dis. 57, 1105–1121 (2017). 28059794 10.3233/JAD-161088 PMC5409043

[11] Al-Ghraiybah N. F. Glial Cell-Mediated Neuroinflammation in Alzheimer’s Disease. Int. J. Mol. Sci. 23, 10572 (2022). 36142483 10.3390/ijms231810572 PMC9502483

[12] Leng F. & Edison P. Neuroinflammation and microglial activation in Alzheimer disease: where do we go from here? Nat. Rev. Neurol. 17, 157–172 (2021). 33318676 10.1038/s41582-020-00435-y

[13] Rajesh Y. & Kanneganti T. D. Innate Immune Cell Death in Neuroinflammation and Alzheimer’s Disease. Cells 11, 1885 (2022). 35741014 10.3390/cells11121885 PMC9221514

[14] van de Haar H. J. Blood-Brain Barrier Leakage in Patients with Early Alzheimer Disease. Radiology 281, 527–535 (2016). 27243267 10.1148/radiol.2016152244

[15] Musaeus C. S., Gleerup H. S., Hasselbalch S. G., Waldemar G. & Simonsen A. H. Progression of Blood-Brain Barrier Leakage in Patients with Alzheimer’s Disease as Measured with the Cerebrospinal Fluid/Plasma Albumin Ratio Over Time. J. Alzheimers Dis. Rep. 7, 535–541 (2023). 37313491 10.3233/ADR-230016 PMC10259070

[16] Lin A. J. In vivo optical signatures of neuronal death in a mouse model of Alzheimer’s disease. Lasers Surg. Med. 46, 27–33 (2014). 24284732 10.1002/lsm.22206 PMC4001800

[17] Wadhwani A. R., Affaneh A., Van Gulden S. & Kessler J. A. Neuronal apolipoprotein E4 increases cell death and phosphorylated tau release in alzheimer disease. Ann. Neurol. 85, 726–739 (2019). 30840313 10.1002/ana.25455 PMC8123085

[18] Oliveira J. F. & Araque A. Astrocyte regulation of neural circuit activity and network states. Glia 70, 1455–1466 (2022). 35460131 10.1002/glia.24178 PMC9232995

[19] Lee Y. F. V. Optogenetic targeting of astrocytes restores slow brain rhythm function and slows Alzheimer’s disease pathology. Sci. Rep. 13, 13075 (2023). 37567942 10.1038/s41598-023-40402-3 PMC10421876

[20] Kuchibhotla K. V., Lattarulo C. R., Hyman B. T. & Bacskai B. J. Synchronous hyperactivity and intercellular calcium waves in astrocytes in Alzheimer mice. Science 323, 1211–1215 (2009). 19251629 10.1126/science.1169096 PMC2884172

[21] Kastanenka K. V. Galea E. A roadmap to integrate astrocytes into Systems Neuroscience. Glia 68, 5–26 (2020). 31058383 10.1002/glia.23632 PMC6832773

[22] Clarke L. E. & Barres B. A. Emerging roles of astrocytes in neural circuit development. Nat. Rev. Neurosci. 14, 311–321 (2013). 23595014 10.1038/nrn3484 PMC4431630

[23] Bouteiller J. M. & Berger T. W. in Encyclopedia of Computational Neuroscience (eds. Jaeger D. & Jung R.) 1–4 (Springer New York, 2015). 10.1007/978-1-4614-7320-6_363-2

[24] Heller J. P. & Rusakov D. A. The Nanoworld of the Tripartite Synapse: Insights from Super-Resolution Microscopy. Frontiers Cell. Neuroscience 11, (2017). 10.3389/fncel.2017.00374 PMC5705901 29225567

[25] McNeill J., Rudyk C., Hildebrand M. E. & Salmaso N. Ion Channels and Electrophysiological Properties of Astrocytes: Implications for Emergent Stimulation Technologies. Front. Cell. Neurosci. 15, 644126 (2021). 34093129 10.3389/fncel.2021.644126 PMC8173131

[26] Kofuji P. & Araque A. G-Protein-Coupled Receptors in Astrocyte-Neuron Communication. Neuroscience 456, 71–84 (2021). 32224231 10.1016/j.neuroscience.2020.03.025 PMC8817509

[27] DeKosky S. T. & Scheff S. W. Synapse loss in frontal cortex biopsies in Alzheimer’s disease: correlation with cognitive severity. Ann. Neurol. 27, 457–464 (1990). 2360787 10.1002/ana.410270502

[28] Takano T., Han X., Deane R., Zlokovic B. & Nedergaard M. Two-photon imaging of astrocytic Ca2 + signaling and the microvasculature in experimental mice models of Alzheimer’s disease. Ann. N Y Acad. Sci. 1097, 40–50 (2007). 17413008 10.1196/annals.1379.004

[29] Tarantini S. Pharmacologically induced impairment of neurovascular coupling responses alters gait coordination in mice. Geroscience 39, 601–614 (2017). 29243191 10.1007/s11357-017-0003-x PMC5745218

[30] Delekate A. Metabotropic P2Y1 receptor signalling mediates astrocytic hyperactivity in vivo in an Alzheimer’s disease mouse model. Nat. Commun. 5, 5422 (2014). 25406732 10.1038/ncomms6422

[31] Åbjørsbråten K. S. Impaired astrocytic Ca2 + signaling in awake-behaving Alzheimer’s disease transgenic mice. Elife 11, e75055 (2022). 35833623 10.7554/eLife.75055 PMC9352348

[32] Lines J. Astrocyte-neuronal network interplay is disrupted in Alzheimer’s disease mice. Glia 70, 368–378 (2022). 34726298 10.1002/glia.24112 PMC8665078

[33] Semyanov A. & Verkhratsky A. Astrocytic processes: from tripartite synapses to the active milieu. Trends Neurosci. 44, 781–792 (2021). 34479758 10.1016/j.tins.2021.07.006

[34] Díaz-Castro B., Robel S. & Mishra A. Astrocyte Endfeet in Brain Function and Pathology: Open Questions. Annu. Rev. Neurosci. 46, 101–121 (2023). 36854317 10.1146/annurev-neuro-091922-031205

[35] Di Castro M. A. Local Ca2 + detection and modulation of synaptic release by astrocytes. Nat. Neurosci. 14, 1276–1284 (2011). 21909085 10.1038/nn.2929

[36] Miller M. R., Lee Y. F. & Kastanenka K. V. Calcium sensor Yellow Cameleon 3.6 as a tool to support the calcium hypothesis of Alzheimer’s disease. Alzheimers Dement. 19, 4196–4203 (2023). 37154246 10.1002/alz.13111 PMC10524576

[37] Webster S. J., Bachstetter A. D., Nelson P. T., Schmitt F. A. & Van Eldik L. J. Using mice to model Alzheimer’s dementia: an overview of the clinical disease and the preclinical behavioral changes in 10 mouse models. Front. Genet. 5, 88 (2014). 24795750 10.3389/fgene.2014.00088 PMC4005958

[38] Jankowsky J. L. Mutant presenilins specifically elevate the levels of the 42 residue β-amyloid peptide in vivo: evidence for augmentation of a 42-specifc γ secretase. Hum. Mol. Genet. 13, 159– 170 (2004). 14645205 10.1093/hmg/ddh019

[39] Eilers P. H. & Boelens H. F. Baseline correction with asymmetric least squares smoothing. Leiden Univ. Med. Centre Rep. 1, 5 (2005).

[40] Abedin M. J., Michelhaugh S. K., Mittal S. & Berdichevsky Y. 3D models of glioblastoma interaction with cortical cells. Front. Bioeng. Biotechnol. 11, 1150772 (2023). 36970613 10.3389/fbioe.2023.1150772 PMC10033518

[41] Galea E. Topological analyses in APP/PS1 mice reveal that astrocytes do not migrate to amyloid-β plaques. Proc. Natl. Acad. Sci. U S A. 112, 15556–15561 (2015). 26644572 10.1073/pnas.1516779112 PMC4697430

[42] Shah D. De Strooper B. Astrocyte calcium dysfunction causes early network hyperactivity in Alzheimer’s disease. Cell. Rep. 40, 111280 (2022). 36001964 10.1016/j.celrep.2022.111280 PMC9433881

[43] Santello M., Toni N. & Volterra A. Astrocyte function from information processing to cognition and cognitive impairment. Nat. Neurosci. 22, 154–166 (2019). 30664773 10.1038/s41593-018-0325-8

[44] Goenaga J., Araque A. & Kofuji P. & Herrera Moro Chao D. Calcium signaling in astrocytes and gliotransmitter release. Front Synaptic Neurosci 15, (2023). 10.3389/fnsyn.2023.1138577 PMC10017551 36937570

[45] Perea G., Navarrete M. & Araque A. Tripartite synapses: astrocytes process and control synaptic information. Trends Neurosci. 32, 421–431 (2009). 19615761 10.1016/j.tins.2009.05.001

[46] Huffels C. F. M., Osborn L. M., Cappaert N. L. M. & Hol E. M. Calcium signaling in individual APP/PS1 mouse dentate gyrus astrocytes increases ex vivo with Aβ pathology and age without affecting astrocyte network activity. J. Neurosci. Res. 100, 1281–1295 (2022). 35293016 10.1002/jnr.25042 PMC9314019

[47] Kelly P. Neuronally Derived Soluble Abeta Evokes Cell-Wide Astrocytic Calcium Dysregulation in Absence of Amyloid Plaques in Vivo. J. Neurosci. 43, 4926–4940 (2023). 37236808 10.1523/JNEUROSCI.1988-22.2023 PMC10312057

[48] Thrane A. S. General anesthesia selectively disrupts astrocyte calcium signaling in the awake mouse cortex. Proc. Natl. Acad. Sci. U S A. 109, 18974–18979 (2012). 23112168 10.1073/pnas.1209448109 PMC3503159

[49] Puri B. K. Calcium Signaling and Gene Expression. Adv. Exp. Med. Biol. 1131, 537–545 (2020). 31646525 10.1007/978-3-030-12457-1_22

[50] Transcription Factor NFAT - an overview | ScienceDirect Topics. at https://www.sciencedirect.com/topics/immunology-and-microbiology/transcription-factor-nfat

[51] Wen A. Y., Sakamoto K. M. & Miller L. S. The Role of the Transcription Factor CREB in Immune Function. J. Immunol. 185, 6413–6419 (2010). 21084670 10.4049/jimmunol.1001829 PMC5519339

[52] Jong Huat T. The impact of astrocytic NF-κB on healthy and Alzheimer’s disease brains. Sci. Rep. 14, 14305 (2024). 38906984 10.1038/s41598-024-65248-1 PMC11192733

[53] Mackiewicz J., Lisek M. & Boczek T. Targeting CaN/NFAT in Alzheimer’s brain degeneration. Front Immunol 14, (2023). 10.3389/fimmu.2023.1281882 PMC10701682 38077352

[54] Gómez-Gonzalo M. NEURON-ASTROCYTE SIGNALING IS PRESERVED IN THE AGEING BRAIN. Glia 65, 569–580 (2017). 28130845 10.1002/glia.23112 PMC5314210

[55] Pavlou M. A. S., Grandbarbe L., Buckley N. J., Niclou S. P. & Michelucci A. Transcriptional and epigenetic mechanisms underlying astrocyte identity. Prog. Neurobiol. 174, 36–52 (2019). 30599178 10.1016/j.pneurobio.2018.12.007

[56] Berridge M. J. The Inositol Trisphosphate/Calcium Signaling Pathway in Health and Disease. Physiol. Rev. 96, 1261–1296 (2016). 27512009 10.1152/physrev.00006.2016

[57] Vincent A. J., Gasperini R., Foa L. & Small D. H. Astrocytes in Alzheimer’s disease: emerging roles in calcium dysregulation and synaptic plasticity. J. Alzheimers Dis. 22, 699–714 (2010). 20847426 10.3233/JAD-2010-101089

[58] Neal M. & Richardson J. R. Epigenetic Regulation of Astrocyte Function in Neuroinflammation and Neurodegeneration. Biochim. Biophys. Acta. 1864, 432–443 (2018). 10.1016/j.bbadis.2017.11.004 PMC5743548 29113750

[59] Nanclares C., Baraibar A. M., Araque A. & Kofuji P. Dysregulation of Astrocyte–Neuronal Communication in Alzheimer’s Disease. Int. J. Mol. Sci. 22, 7887 (2021). 34360652 10.3390/ijms22157887 PMC8346080

[60] Araque A. Gliotransmitters Travel in Time and Space. Neuron 81, 728–739 (2014). 24559669 10.1016/j.neuron.2014.02.007 PMC4107238

[61] Lines J., Martin E. D., Kofuji P., Aguilar J. & Araque A. Astrocytes modulate sensory-evoked neuronal network activity. Nat. Commun. 11, 3689 (2020). 32704144 10.1038/s41467-020-17536-3 PMC7378834

[62] Metea M. R. & Newman E. A. Glial Cells Dilate and Constrict Blood Vessels: A Mechanism of Neurovascular Coupling. J. Neurosci. 26, 2862–2870 (2006). 16540563 10.1523/JNEUROSCI.4048-05.2006 PMC2270788

[63] Mulligan S. J. & MacVicar B. A. Calcium transients in astrocyte endfeet cause cerebrovascular constrictions. Nature 431, 195–199 (2004). 15356633 10.1038/nature02827

[64] Girouard H. & Iadecola C. Neurovascular coupling in the normal brain and in hypertension, stroke, and Alzheimer disease. J. Appl. Physiol. (1985). 100, 328–335 (2006). 16357086 10.1152/japplphysiol.00966.2005

[65] Iadecola C. Neurovascular regulation in the normal brain and in Alzheimer’s disease. Nat. Rev. Neurosci. 5, 347–360 (2004). 15100718 10.1038/nrn1387

[66] Zlokovic B. V. Neurovascular pathways to neurodegeneration in Alzheimer’s disease and other disorders. Nat. Rev. Neurosci. 12, 723–738 (2011). 22048062 10.1038/nrn3114 PMC4036520

[67] Haydon P. G. & Carmignoto G. Astrocyte Control of Synaptic Transmission and Neurovascular Coupling. Physiol. Rev. 86, 1009–1031 (2006). 16816144 10.1152/physrev.00049.2005

[68] Nycz B. & Mandera M. The features of the glymphatic system. Auton. Neurosci. 232, 102774 (2021). 33610009 10.1016/j.autneu.2021.102774

[69] Iliff J. J. Paravascular Pathway Facilitates CSF Flow Through the Brain Parenchyma and the Clearance of Interstitial Solutes, Including Amyloid β. Science Translational Medicine 4, 147ra111–147ra111 (2012). 10.1126/scitranslmed.3003748 PMC3551275 22896675

[70] Thrane A. S. Critical role of aquaporin-4 (AQP4) in astrocytic Ca2 + signaling events elicited by cerebral edema. Proc. Natl. Acad. Sci. U S A. 108, 846–851 (2011). 21187412 10.1073/pnas.1015217108 PMC3021020

[71] Pedersen T. J., Keil S. A., Han W., Wang M. X. & Iliff J. J. The effect of aquaporin-4 mis-localization on Aβ deposition in mice. Neurobiol. Dis. 181, 106100 (2023). 36990365 10.1016/j.nbd.2023.106100

[72] Yamada K. Multifaceted Roles of Aquaporins in the Pathogenesis of Alzheimer’s Disease. Int. J. Mol. Sci. 24, 6528 (2023). 37047501 10.3390/ijms24076528 PMC10095057

